# Successful treatment of tracheal stenosis by rigid bronchoscopy and topical mitomycin C: a case report

**DOI:** 10.1186/1757-1626-3-2

**Published:** 2010-01-04

**Authors:** Jyi Lin Wong, Siew Teck Tie, Bohari Samril, Chee Lun Lum, Mohammad Rizal Abdul Rahman, Jamalul Azizi Abdul Rahman

**Affiliations:** 1 Department of Respiratory Medicine, Queen Elizabeth Hospital, Kota Kinabalu, Sabah, Malaysia; 2 Department of Othorhinolaryngology, Queen Elizabeth Hospital, Kota Kinabalu, Sabah, Malaysia; 3 Department of Anaesthesiology, Queen Elizabeth Hospital, Kota Kinabalu, Sabah, Malaysia

## Abstract

Tracheal stenosis is a known complication of prolonged intubation. It is difficult to treat and traditional surgical approach is associated with significant risk and complications. Recurrent stenosis due to granulation tissue necessitates repeated procedures. We describe a case of short web-like tracheal stenosis (concentric membranous stenosis less than 1 cm in length without associated cartilage damage) managed by a minimally invasive thoracic endoscopic approach. Topical application of Mitomycin C, a potent fibroblast inhibitor reduces granulation tissue formation and prevents recurrence.

## Introduction

MacEwen first reported endotracheal intubation for anesthesia in 1880 [1]. Lindholm reported injuries to the larynx and trachea after intubation in 1969 [2]. Despite advancement and the use of high volume, low pressure cuffed tubes, tracheal stenosis is not an uncommon complication of endotracheal intubation. In one prospective study of critically ill patients, 11% of patients who were intubated with high volume, low pressure cuffed tubes developed tracheal stenosis [3]. Endotracheal tube causes pressure injury to the glottis and may result in severe commissural scarring that is difficult to treat.

Although there have been reports of successful treatment of tracheal stenosis with steroid regimens [4,5], the mainstay of treatment for symptomatic lesions is surgical. Various surgical methods have been described including anterior cricotracheal splitting, laryngofissure creation with anterior lumen augmentation, resection or end-to-end anastomosis [6-8], but they are not without risks. Tracheal reconstruction requires major surgery, with a mortality of about 3% [9,10]. Rigid bronchoscopy with tracheal dilatation and stenting has been described as some of the treatment methods for less serious lesions [11]. Relapses are relatively frequent, making it the principal long-term problem with this method of treatment [12,13].

## Case presentation

A 30-year-old Malaysian woman who had bilateral upper lobe lung bullae underwent bullectomy in May 2008 for rapidly enlarging bullae causing respiratory compromise. Post-operatively, she was intubated for 6 days. Upon trial of extubation at day 6, she developed shortness of breath. She was re-intubated for another 5 days before extubation was successful and she was discharged 20 days post operatively.

**Figure 1:**
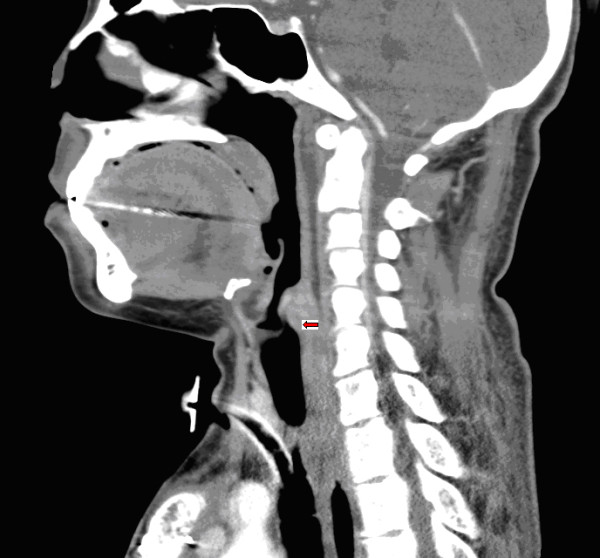
**Sagittal and coronal view of the CT scan showing a short segment stenosis just above the tracheostomy**.

She was well for the next one month until July 2008 when she developed a sudden onset of shortness of breath and stridor. Intubation attempt with a 4 mm endotracheal tube was unsuccessful leading to emergency tracheostomy. Flexible bronchoscopy in the operating room revealed a membranous web-like concentric stenosis without cartilage involvement 3 cm below the vocal cords. Bronchoscopy through the tracheostomy showed normal distal airways. A neck CT scan confirmed the presence of a short segment tracheal stenosis (less than 1 cm) [figure 1].

In August 2008, she underwent rigid bronchoscopy using a 12 mm Dumon rigid tracheal tube. This was followed by examination using a small diagnostic flexible bronchoscope via the rigid bronchoscope. A pin hole stenosis of the trachea 3 cm distal to the vocal cords was noted with areas of granulation tissue. We failed to pass the flexible bronchoscope through the stenosis.

**Figure 2:**
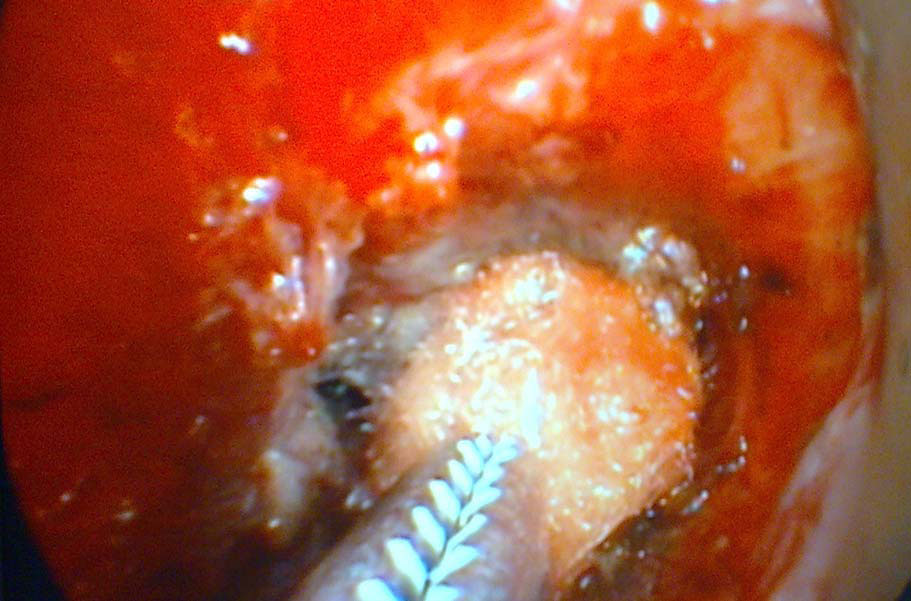
**A pledget soaked with Mitomycin C applied via rigid forceps to the stenotic area**.

**Figure 3:**
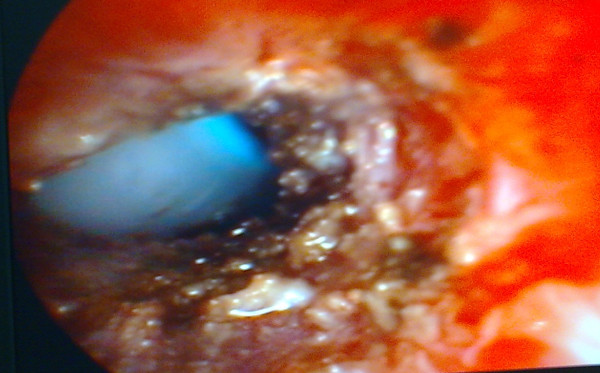
**The appearance of the stenotic area immediately after application of Mitomycin C**.

Initially argon plasma coagulation (APC) was used to devitalize the granulation tissues surrounding the stenosis. Rigid forceps and cryoprobe were used to remove the necrotic tissues. The APC and cryo probes were inserted through the side-port of the Dumon rigid bronchoscope instead of through the working channel of the flexible bronchoscope to minimize damage to the working channel of the flexible bronchoscope. Bougies was then used to dilate the stenotic area in incremental size to a final size of 1 cm. Pledgets soaked with diluted Mitomycin C (concentration 0.2 mg/ml) were introduced using rigid forceps through the rigid bronchoscope to the raw surface of the mucosa and direct pressure applied via the rigid forceps [figure 2]. Pressure was applied for 2 minutes until blanching of the mucosa was seen. The procedure was repeated circumferentially until all mucosa was treated. A total of 4 applications or 8 minutes of contact time was needed [figure 3]. Hemostasis was then secured with pledgets soaked with adrenaline and the rigid bronchoscope was removed. The whole procedure took 110 minutes.

**Figure 4:**
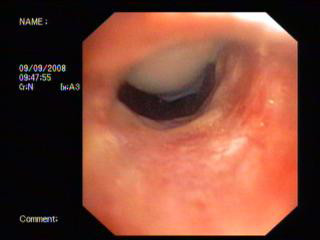
**The appearance of the stenotic area 2 weeks after the procedure**.

Intravenous dexamethasone 8 mg tds was given for 3 days. At 2 weeks, a flexible bronchoscopy showed a patent airway and she was weaned off the tracheostomy tube after she tolerated spigotting for 48 hours [figure 4].

**Figure 5:**
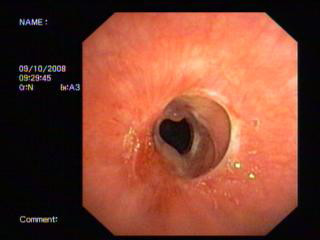
**The appearance of the stenotic area after one month: Noted there was a small granulation tissue over the anterior surface of the mucosa, but a flexible bronchoscope could still be passed through easily**.

**Figure 6:**
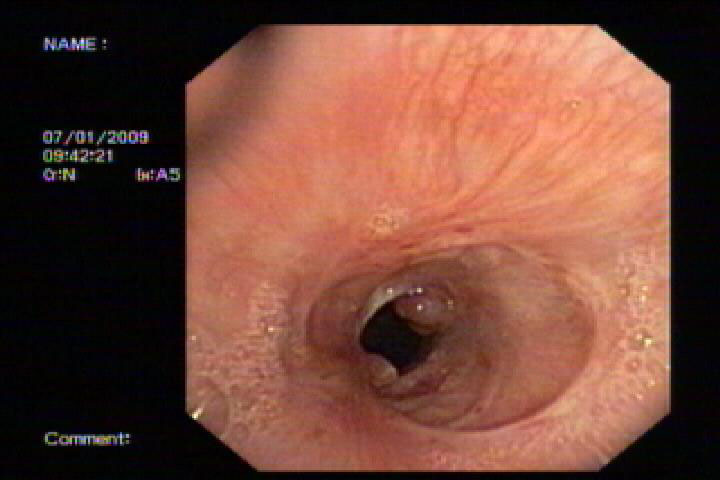
**The appearance of the stenotic area after 4 months: Noted the granulation tissue was still present but not enlarging and a flexible bronchoscope could still be passed through easily**.

Repeat flexible bronchoscopy was done at 1 month [figure 5] and then 4 month [figure 6] after the procedure. The trachea remained patent. A small granulation nodule was seen on the anterior wall of the trachea but a 6 mm therapeutic flexible bronchoscope could easily be passed through the stenosis.

There is no significant worsening of the narrowing between 1 month and 4 month. She did not complain of stridor or difficulty in breathing and was back to work.

## Discussion

The optimal treatment of tracheal stenosis remains undefined. Traditionally, tracheal stenosis has been managed by thoracic and othorhinolaryngology surgeons. Endoscopic procedures are usually performed as a bridge to definitive surgical intervention. With the development of interventional pulmonology field in the last 20 years, definitive management of tracheal stenosis using minimally invasive endoscopic methods became a possibility. Collaboration between pulmonologists and surgeons has become increasingly important to help define the best method of management for these patients.

Endoscopic treatment had been shown to be useful, especially in patients who are deemed high risk and too unwell for reconstructive surgery. One of the main drawbacks of endoscopic treatment and surgery is the risk of recurrence of trachea stenosis due to granulation and fibrotic tissue. Studies have shown that most of the recurrence of tracheal stenosis occurs within one to three months after the procedure [12,14], and use of Mitomycin C has been reported in case studies to reduce the rate of recurrence [13,15]. Mitomycin C which is isolated from *Streptomyces caepitocus *acts as a bifunctional alkylating agent cross-linking DNA thereby inhibiting DNA synthesis. Both in vitro and in vivo, Mitomycin C has been proven to be a potent inhibitor of human fibroblasts at concentrations of 0.04 mg/L. It has been used with some success in inhibiting the vigorous granulation response noted after airway injury in animal models and pediatric patients. It has also been used in ophthalmology to treat glaucoma and pterygium.

In this case, we describe a successful management of a short web-like tracheal stenosis via rigid bronchoscopy by interventional pulmonologists with collaboration from othorhinolaryngology surgeons. APC appears to be an effective coagulation method. The use of APC prior to granulation tissue removal allows for easy control of bleeding. The application of cryoprobe to remove granulation tissues also minimizes trauma to the trachea.

Nd:YAP laser would be an interesting alternative to APC for coagulation but it was not available to us at the time. The presence of othorhinolaryngology surgeons during the procedure who have a good knowledge of local airway anatomy allows identification of structures which are distorted by granulation tissues. This prevents the formation of false tract and accidental perforation of the trachea.

In skilled hands, the use of a suspension laryngoscope would be an alternative to rigid bronchoscope in this case. Suspension laryngoscope is ideal if the lesion is either at the vocal cords or just below the vocal cords (subglottic). However, suspension laryngoscope needs to be used with a caveat that there may be a higher risk of irritation and injury to the vocal cords during insertion of surgical instruments to reach the target area which was 3 cm below the vocal cords. Furthermore, the use of a suspension laryngoscope will require jet-ventilation in order to ventilate the patient. The use of rigid bronchoscope allows us to introduce surgical instruments multiple times to the target area with minimal risk of irritating or injuring the vocal cords.

The application of topical Mitomycin C is made possible with the rigid forceps which allows application of sufficient force to the airway mucosa to allow proper application of Mitomycin C. It is important to apply Mitomycin C with sufficient force and contact time to allow Mitomycin C to work. With a contact time of 8 minutes, there is evidence of only minimal granulation tissue or scar tissue after 4 months. Topical Mitomycin C also appears to be safe and there is no detectable myelosuppression.

## Conclusion

Topical Mitomycin C is a useful adjunct in the management of short concentric membranous stenosis which does not involve the cartilage. It reduces granulation tissue and prevents recurrence. Further randomized controlled studies are necessary to explore the possibility of this treatment method in the algorithm for the management of tracheal stenosis. This may obviate the need for invasive reconstruction surgery and repetitive procedures.

## Consent

Written informed consent was obtained from the patient for publication of this case report and accompanying images. A copy of the written consent is available for review by the Editor-in-Chief of this journal

## Competing interests

The authors declare that they have no competing interests.

## Authors' contributions

JLW prepared the final copy of the manuscript. STT performed rigid bronchoscopy and the initial bougie dilatation. BS prepared mitomycin C and reviewed articles on the dosage and safety of mitomycin C before application. CLL applied mitomycin C and guided the team on the anatomy of the tracheal stenosis. MR was responsible for the administration of general anaesthesia to the patient during the procedure. ARJA initiated the idea of rigid bronchoscopy and mitomycin C application, supervised the whole procedure and reviewed the final manuscript. All authors have read and approved the final manuscript
